# Divergent beliefs about food safety and affordability in Nigeria

**DOI:** 10.1016/j.gfs.2024.100753

**Published:** 2024-06

**Authors:** Lenis Saweda O. Liverpool-Tasie, Ayala Wineman, Danielle Resnick

**Affiliations:** a Department of Agricultural, Food, and Resource Economics, Michigan State University, USA; b International Institute of Tropical Agriculture (IITA), Nigeria; c Global Child Nutrition Foundation, USA; d International Food Policy Research Institute (IFPRI), USA

**Keywords:** Best-worst scaling, Food safety, Food systems, Nigeria, Policy beliefs, Value chains

## Abstract

Access to safe, affordable diets is paramount for improved nutritional outcomes. Yet, how do stakeholders perceive the binding constraints and requisite policy actions to increase food safety and affordability? Focusing on Nigeria, this paper uses best-worst scaling techniques applied to a survey of 200 government and agrifood system stakeholders to examine their policy beliefs on safety and affordability vis-à-vis the vegetable and fish value chains. We find that divergence among stakeholders is greater for food safety than affordability. While antibiotics overuse and toxin exposure, lack of knowledge, and weak legislation were identified by different stakeholders as the binding constraints for food safety, high costs of inputs and infrastructure, as well as security threats, were seen as common challenges for affordability across most, though not all, stakeholders for both value chains. Overall, the paper highlights the importance of beliefs in the agrifood system policymaking process and emphasizes the need to explore not only the existence but also the source of divergent beliefs among policy actors in greater depth.

## Highlights

• Divergent policy beliefs are problematic for complex issues, like food safety and affordability.
• Food safety and affordability of healthy foods are challenges in countries like Nigeria.
• Two hundred stakeholders in Nigeria's fish and vegetable value chains were surveyed.
• Best-worst scaling methods reveal conflicting policy beliefs about safety and affordability.
• Reconciling divergent beliefs is critical for policy prioritization.

## 1 Introduction

How do divergent policy beliefs affect policy prioritization for achieving healthy food systems? In the context of increasingly constrained financial resources, low and middle-income countries (LMICs) need to prioritize where they allocate scarce budgets to improve their populations’ access to safe, nutritious foods (Diaz-Bonilla et al., 2023). However, prioritization requires policymakers to have a shared understanding of the main challenges to be overcome, what solutions are required, and how solutions should be sequenced. Moreover, these understandings need to resonate with affected stakeholders for policy interventions to be seen as legitimate. Achieving these conditions can be particularly difficult for policy issues that are multi-scalar and that require not only inter-governmental and cross-jurisdictional coordination, but also cooperation of multiple non-governmental actors, including the private sector, civil society, and consumers.

In this study, we examine the existence and implications of divergent policy beliefs through a specific focus on food safety and affordability. Food safety is a quintessential multi-scalar issue that involves national regulatory frameworks, subnational enforcement, coherence with international standards, respect for traditional knowledge, and oversight of diverse actors in the food value chain (Yasuda, 2018). For LMICs, food safety management is further complicated by the co-existence of informal and formal food supply channels (Jaffee et al., 2018). At the same time, limited budgets, a dearth of skilled human resources, and insufficient technology (e.g. laboratories) often means that food safety agencies in LMICs typically must be selective in their food safety efforts (Henson et al., 2023). Food costs also rely on multi-scalar and multi-stakeholder coordination; the costs encountered by consumers reflect the cumulative impact of input availability, trade decisions, fiscal policies, infrastructure investments, and regulatory policies. Moreover, affordability complicates concerns over safety; fresh fruits and vegetables and fish are viewed as integral parts to a healthy diet (Willett et al., 2019). Yet, especially for the poorest households, such products may not be affordable (Hirvonen et al., 2020) or are most economical when purchased in informal markets where food safety issues tend to be most pronounced (Henson et al., 2023).

While protecting the safety of healthy foods and improving their affordability is rarely disputed, how different actors perceive what should be prioritized and who is responsible is rarely studied in LMICs. Instead, studies focus on consumer perceptions of food safety (Mergenthaler et al., 2009; Nordhagen et al., 2022; Traoré et al., 2018; Zanetta et al., 2022), food handlers’ preparation techniques, hygiene, and knowledge about food safety risks (Manes et al., 2023), or consumer beliefs about the costs and composition of a healthy diet (Hill et al., 2016; Lusk, 2019). By contrast, we examine the most binding constraints to improving food safety and food affordability as perceived by a range of stakeholders engaged in the agrifood policy system, either as a value chain participant, policy formulator and implementer, financer, advocate, or part of the epistemic community informing science-based decisions (e.g. researchers).

We focus on Nigeria, where the economic burden of foodborne diseases, measured by the costs of mortality and morbidity, was estimated at over USD 6 billion in 2016—higher than any other country in Africa and the fourth highest in the world (Jaffee et al., 2019). The cost of a healthy diet is likewise problematic in the country; recent analysis finds that it is costlier to meet the dietary recommendations for vegetables, protein-rich foods like fish, and dairy in Nigeria than other food groups (Mekonnen et al., 2021). We implemented a survey with 200 knowledgeable stakeholders to examine perceptions of food safety and affordability challenges. Our study focuses on the domestic supply chains for the studied products given the dominant role of domestic food supply chains in meeting the food needs in Nigeria and other African countries (Liverpool-Tasie et al., 2021b; Reardon et al., 2019). Using best-worst scaling (BWS), a methodological approach used to uncover stakeholders’ policy priorities in both an ordinal and cardinal manner, we uncover how different stakeholder groups perceive food safety and affordability challenges for fish and vegetables and their preferred policy responses.

We find several areas of notable divergence. For fish, federal government stakeholders are most likely to see lack of food safety knowledge by agrifood system actors as the biggest challenge to food safety. By contrast, state-level actors view weak food safety legislation as most problematic, while farmers and industry see fish treated with antibiotics and affected by toxins as top concerns. With respect to affordability of fish, the federal government sees the high cost of infrastructure, especially storage facilities, as the top barrier while almost all other stakeholders prioritize the high cost of inputs such as feed and equipment. When asked to consider the best and worst options for improving the affordability and/or safety of fish, farmers disproportionately prioritize receiving subsidies or cash transfers even as most others see that enhancing productivity through research and training of fishers and fish farmers would be the best option.

For vegetables, the federal government again places the most emphasis on agrifood system actors’ insufficient knowledge, even as other stakeholders also view lack of infrastructure, weak food safety legislation, and a lack of specific guidelines in informal markets as among the most acute bottlenecks for improved food safety. On vegetable affordability, all stakeholder categories believe that high input costs are the main challenge but then there is substantial divergence between stakeholders and other possible challenges; those belonging to the federal government predominantly prioritize the availability and cost of electricity as the main constraint, researchers identify poor roads, and industry actors point to security challenges. Similar to fish, vegetable farmers believe that subsidies and cash transfers would help improve the affordability and/or safety of vegetables.

The next section examines scholarship on the role of policy beliefs in food systems. This is followed by an overview of food safety and affordability challenges in Nigeria. Subsequently, we elaborate on our data and methods before turning to our empirical findings, discussion, and conclusion.

## 2 The salience of policy beliefs

Policymaking is often the outcome of multiple, intersecting dynamics, including institutional structures, power struggles across ministries, partisan ideologies and electoral cycles, modalities of collective action among interest groups, and the skills and capacity of public and private sector implementing agents (Resnick et al., 2018; Resnick and Swinnen 2023). In this paper, we focus specifically on the role of policy beliefs in the policymaking process since they shape the preferences of different interest groups and thereby influence their positions on different types of interventions. When those beliefs diverge significantly, policy options can become skewed towards the beliefs of the stakeholders with the most power, measured either as those with the most control over budget decisions, with the most visibility and voice, or those who are institutionally and legally prescribed with veto powers to make decisions (Tsebelis, 2002).

Policy beliefs have gained growing prominence in public policy studies and increasingly are viewed as critical to understand when and why policy change occurs (Béland and Cox, 2010; Blyth, 2015; Hall, 1993). Beliefs capture an individual's interpretation about cause-and-effect relationships and their normative assumptions (Jervis, 2006), and they serve as a heuristic tool through which empirical analysis is filtered and acted upon.

One seminal application of policy beliefs is encapsulated by the advocacy coalition framework (ACF), which underscores that within each policy sub-system (e.g. nutrition, education, agriculture), there are sets of actors whose shared policy beliefs drive coalition formation; changes in a coalition's beliefs influences policy changes (Sabatier, 1988; Sabatier and Weible, 2007). One of the three types of beliefs are “core beliefs,” which capture similarities and differences in how stakeholders perceive the seriousness of a problem or the causes of the problem (Jenkins-Smith et al., 2014; Rietig, 2018). Divergence over policy core beliefs is most problematic for policy prioritization because, while there may be consensus about the need for action, there is disagreement over what is the binding constraint and how actions should be sequenced.

These beliefs can derive from many different sources, including occupational position, familial influences, and education. For instance, “street level bureaucrats” who engage in policy implementation may observe different challenges on the ground than their government counterparts who focus on policy formulation. Mogues and Olofinbiyi (2020) find that across the three tiers of government in Nigeria, technical bureaucrats at the state level have very different ideas than their elected national colleagues about how budgets could be allocated to improve agricultural productivity; they attribute this divergence to information asymmetries across the different tiers. The policy feedback literature emphasizes that individuals’ beliefs, interests, and preferences are influenced by how extant policies are enacted and experienced on the ground (Campbell, 2012; Lynch and Myrskylä, 2009; Mettler and Soss, 2004). For instance, if a government failed to implement a promised policy program as intended, this creates a negative feedback effect that undermines trust in other policy arenas. Such distrust can be highest for policy issues that are complex for stakeholders to understand (legislation and regulations), low visibility (e.g. investments in research and development), or for which a government has demonstrated a repeated inability to tackle (Batley and Mcloughlin, 2015; Mogues, 2015). For instance, Kyle (2018) suggests that low trust is one reason why citizens favor costly input subsidies that only generate short-term benefits rather than larger-scale investments that would broaden growth and transformation. Other literature emphasizes the role of socioeconomic status and cultural upbringing in driving policy beliefs (Ballew et al., 2020; Saint-Paul 2010; Sherman et al., 2022).

Research using the ACF and examining policy beliefs around subsidies and ultra-processed foods in LMICs is gaining prominence (Harris, 2019; Mockshell and Birner, 2020; Mockshell and Ritter 2023). Yet, to our knowledge, there is no analysis of belief divergence related to food safety and affordability issues. As such, we build on extant scholarship to argue that policy beliefs on food safety and affordability may not only vary according to one's position in the policy process but also according to the value chain under consideration. Moreover, reconciling divergent beliefs across groups is critical for policy prioritization when multiple challenges exist simultaneously. For instance, analyses of food safety tend to identify a range of needed interventions, from improved infrastructure, to better government capacity for surveillance, to training of informal traders about proper handling techniques (GFSP, 2019). While all of these are critical, they are not all financially feasible and therefore, identifying where beliefs diverge and converge can assist with prioritization. Areas of convergence are likely to be the “low hanging” options for reform in the short run, while those with the greatest polarization may require more time to reconcile.

## 3 The Nigerian context

Our analysis of divergent beliefs focuses on food safety and affordability of fish and vegetables in Nigeria. Like other LMICs, Nigeria is facing dietary changes due to increased incomes, urbanization, and population growth that have triggered a dynamic food supply response, often by numerous micro, small, and medium-sized enterprises, in a context of poor infrastructure and regulatory systems (Jaffee et al., 2018; Nordhagen et al., 2023). Fish and vegetables are ideal candidates for this analysis. First, both sub-sectors have expanded in Nigeria due to changing consumption patterns. Fish accounts for 35% of the budget allocated to animal-source foods in the average household (Liverpool-Tasie et al., 2021a). In addition, practically all Nigerian households consume some vegetables in a typical week (Parkhi et al., 2023); Wineman and Liverpool-Tasie, 2022). Yet, due to price inflation and currency depreciation, the affordability of such foods is under stress (Olayinka et al., 2023) and especially out of reach in rural areas, northern Nigeria and amongst the poorest households (Mekonnen et al., 2021).

Second, the fish and vegetable value chains face important food safety challenges (Liverpool-Tasie et al., 2023; Wineman and Liverpool-Tasie, 2022; Nordhagen et al., 2022), with studies finding dangerous bacteria and toxins in both raw vegetables and smoked fish in Nigerian markets (Nordhagen et al., 2023; Grace et al., 2018). During production, vegetable contaminants could be biological (e.g., viruses or bacteria), chemical (e.g., pollutants in water and soil), or physical (e.g., metals) and occur because vegetables are typically cultivated in open environments (Kahramanoglu, 2017; Yen et al., 2018). Among these contaminants, bacterial hazards are the biggest contributor to the burden of disease in Africa (Havelaar et al., 2015). In Nigeria, where about half of fish come from open water and half from aquaculture, pollutants in water bodies and use of contaminated water or feed are significant production level risks (Uzomah et al., 2021; Obadina 2023a). Post capture/harvest handling, storage, and transportation procedures can also result in contamination (Olaimat and Holley, 2012; Obadina, 2023a). Third, while vegetables and fish are both highly perishable, they have different configurations and peculiarities with implications for food safety. For example, vegetables are often consumed in raw or lightly cooked forms (e.g. in salads or raw accompaniments to food, as ready to eat carrots, and in steamed vegetables) while fish is often consumed in processed form as smoked or dried fish. When fish is processed using firewood and/or charcoal, it exposes consumers to carcinogenic compounds (Uzomah et al., 2021, World Health Organization, 2021).

These challenges are exacerbated by weak food safety infrastructure and regulation (Ezirigwe, 2018; Omojokun, 2013; Ukwueze, 2019), and a lack of coherence in government policy. At the federal level, there are over a dozen Ministries, Departments, and Agencies that have some mandate over food safety policy (Resnick et al., 2023). While food safety policies in Nigeria are designed at the National/Federal level, implementation is undertaken by the three tiers of government: Federal, State, and Local Government. Recent reviews of Nigeria's food safety policies note poor within-government coordination in terms of design and execution, and limited capacity for implementation (Obadina, 2023b; Okoruwa and Onuigbo-Chatta, 2021).

## 4 Data and methods

### 4.1 Data

This study leverages primary data collected from 200 agrifood stakeholders in Nigeria in May–July 2022. A survey questionnaire captured basic information on the respondents and general perceptions of the food system with a focus on the fish and vegetable value chains. By focusing on two value chains that face different constraints to safety and affordability, we can better uncover whether policy belief divergence is specific to the nature of a particular commodity or reflective of the differences in experience, information, and responsibilities among disparate agrifood system actors. The survey was mostly administered online, though a small number of respondents completed the survey on paper (34) or verbally over the phone (27).

Three approaches were used to identify respondents. First, all stakeholders who attended the launch of a research project aiming to support African MSMEs to provide safe and nutritious food were invited to participate in the survey. These included representatives of research/academia, industry, production, government, civil society, and development partners. Second, invitations were extended to professional and personal networks of those affiliated with the research project. Third, potential respondents were identified through extensive online research. Effort was made to ensure geographic representation from both the north and south of Nigeria, as well as representation across different food products (e.g., fish and vegetables) and a wide set of stakeholder groups (e.g., government representatives from both state and federal levels).

Among the final sample, 45.5% and 54.5% of respondents were from southern and northern Nigeria, respectively (Table 1) and this approximates national population figures from the National Bureau of Statistics (NPC and NBS, 2016). About one third (34.5%) of respondents were representatives of research/academia, 23% were farmers, 22.5% were representatives of industry/the private sector, 11% were representatives of government, and 6% were representatives of civil society or development partners. The survey captured a vast set of perspectives from those working directly within each value chain, those indirectly involved, as well as those making policy decisions about it. While these methods of outreach mean our results are not necessarily representative of the full universe of agrifood stakeholders in Nigeria, they nonetheless allow for substantial variety in stakeholder groups more directly relevant to food supply and distribution.

**Table 1:** Stakeholder groups represented in the sample (number of respondents).

	North	South	Total
Civil society organization	3	1	4
Donor/Development partner	7	1	8
Farmer	27	19	46
Government (Federal level)	9	3	12
Government (State level)	2	9	11
Industry/Private sector	25	20	45
Other	3	2	5
Research/Academia	33	36	69
Total	109	91	200

Just over half (54.5%) of respondents were men, and most respondents had over 10 years of formal schooling (90.7%). As the current rate of secondary school net enrollment in Nigeria is just 66% (UNESCO Institute of Statistics UIS, 2022), our sample is heavily skewed towards higher levels of education. Variations in education levels are often found to be a factor that explains variations in the level of technical knowledge about food safety (Chengat Prakashbabu et al., 2020). However, the relatively high level of education among our sample allows us to probe if different stakeholders of similar education level have different perceptions about the availability of safe and affordable food. Over one-fifth (21.5%) of respondents were involved in the fish value chain for their livelihood, 30.5% were directly engaged in the value chains for vegetables or fruits, 15 % were involved in both, and 33% did not work within either the fish or horticulture value chains but worked broadly on food safety, nutrition, and agrifood systems in the country.

### 4.2 Methods

A best-worst scaling (BWS) approach to eliciting preferences was used at several points in the survey. BWS entails presenting respondents with a set of multiple policy options and asking them to indicate which one is ‘best’ (most preferred or important) and which one is ‘worst’ (least preferred or important). Respondents each complete a series of such choice sets, and their choices are used to construct cardinal rankings of the policies (Finn and Louviere 1992; Lusk and Briggeman 2009; Wolf and Tonsor 2013).

BWS offers three major advantages over traditional methods used to assess policy beliefs and preferences, such as approve/disapprove or Likert scale-type questions asked policy-by-policy. First, with these traditional approaches, respondents are not required to make tradeoffs among problems or policies – they can rank all policies as “very important” or “very problematic.” By contrast, BWS explicitly requires respondents to make tradeoffs (Lusk and Briggeman 2009). Second, the scales for rating-based methods such as Likert scales are subjective, such that on a scale of one to five, a four could mean something different to different respondents. There is no such subjectivity with BWS, as respondents are choosing the extremes – the best and worst policy options (Lusk and Briggeman 2009). Third, respondents more easily and consistently choose the extremes in each of a series of choice sets (as in BWS) than when they rank four or more options in a single choice set, as in traditional ranking methods (Marley and Louviere, 2005). Overall, BWS allows priorities to be captured in both an ordinal and cardinal manner; we can rank the listed items and also discern the intensity with which items are more or less preferred.

Although BWS has been used widely in agriculture and resource management research in Europe and North America (Atta and Micheels, 2020; Jones et al., 2013; Scarpa et al., 2011; Stone et al., 2018), its application thus far in LMICs contexts has been limited (for exceptions, see Mason et al., 2019; Maredia et al., 2022). Moreover, most agrifood studies that employ BWS have looked either at producers (Atta and Micheels, 2020, Ortega et al., 2015, Wolf and Tonsor, 2013)or consumers (Loose and Lockshin, 2013; Lusk and Briggeman, 2009). To our knowledge, this approach has not previously been used to uncover policy beliefs for a diverse range of actors involved in the policy process, whether at a national or sub-national level.

To gather stakeholders' views on challenges in Nigeria's fish and vegetable value chains, respondents were asked to consider a list of items and select those they believed to be most and least important. These responses are analyzed by assigning a value of +1 to options selected as most important, −1 to options selected as least important, and 0 to options that were not selected. In our results, these values are sometimes summed over the sample to discern how the group collectively ranks the various options; alternatively, these values are sometimes averaged within a given subsample to compare the ordering and intensity of prioritizations across different respondent categories. Results for various subsamples are presented, and t-tests and chi-squared tests are used to understand whether any differences are statistically significant.

## 5 Findings

### 5.1 General perceptions of safety and affordability

**Fig. 1:**
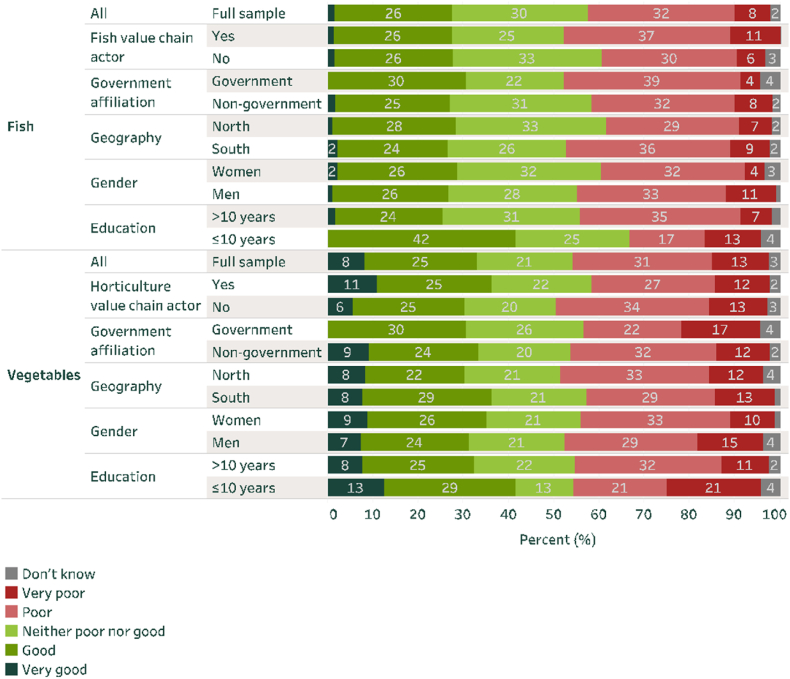
Status of the food safety of fish and vegetables Note: Note: Sample size is as follows: Full sample (200); Government (23); Non-government (177), North (109), South (91), Women (91), Men (109), >10 years education (174), ≤10 years education (24).

Source: RSM2SNF Nigeria Stakeholder Perceptions Survey 2022

Before presenting the BWS findings, Fig. 1 highlights respondents’ overall views on food safety and affordability for fish and vegetables. Overall, 40% and 44% of respondents considered food safety to be poor or very poor for fish and vegetables, respectively (Fig. 1). Across regions, perceptions were similar for vegetables but different for fish. This difference across regions may reflect geographic differences in engagement in the fish subsector and thus different levels of knowledge of fish safety, whereas vegetables are more uniformly known. A majority of fish production in Nigeria occurs in the south (World Health Organization, 2021), and while practically all Nigerian households (in the north and south) consume some vegetables in a typical week, just about 50% of households in the north consume fish compared to 90% in the south (Parkhi et al., 2023; Liverpool-Tasie et al., 2021a).

When perceptions of food safety are disaggregated by gender, perceptions are more similar for vegetables than fish. This is driven by a slight (though only marginally significant) difference in the share that view the safety of fish to be very poor (11% for males and 4% for females) (χ^2^ = 2.788, P = 0.095). A possible explanation is that men are more involved in fish production and/or processing and are thus more familiar with some food safety issues. When perceptions are disaggregated by whether the respondent is somehow directly engaged with the value chain in question, we find that 48% of those in the fish value chain view food safety for fish to be poor or very poor, whereas this is value is much lower (at 36%) for those not in the fish value chain.

**Fig. 2:**
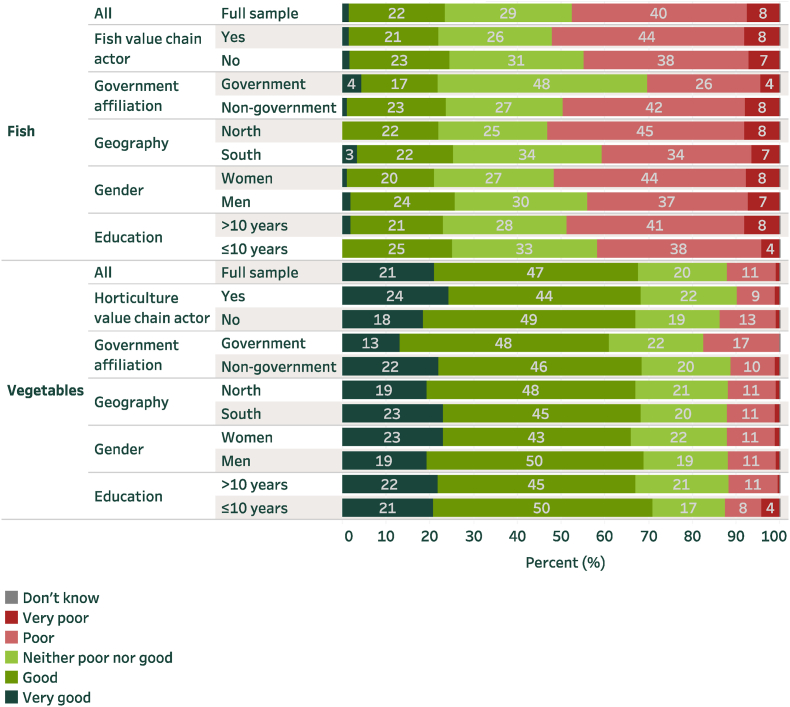
Status of the affordability of fish and vegetables Note: Sample size is as in Fig. 1.

Regarding affordability, there is variance in perspectives between vegetables and fish (Fig. 2). Overall, vegetables are viewed as much more affordable than fish, with 58% of all respondents considering vegetable affordability to be either good or very good. For fish, this value is just 23% (χ2 = 6.710, P = 0.010). Females are slightly (though not significantly) more likely than males to view fish affordability as poor or very poor (χ2 = 1.152, P = 0.283), whereas no such gender difference is evident for vegetables. Across regions, fish is considered more affordable in the south than the north. As more fish production occurs in the south, affordability in the north may be affected by local scarcity.

### 5.2 Sub-group views on food safety

Until this point, we have used Likert scales to discern perceptions of the food system. As noted in section 4.2, a BWS approach to elicit priorities offers greater leverage to probe beliefs, enabling us to gauge both the order and intensity with which items are more or less preferred. Along these lines, respondents were next asked to consider a list of six challenges related to the safety and affordability of fish or vegetables (separately) and to select the two that were most and least serious/important.

**Fig. 3:**
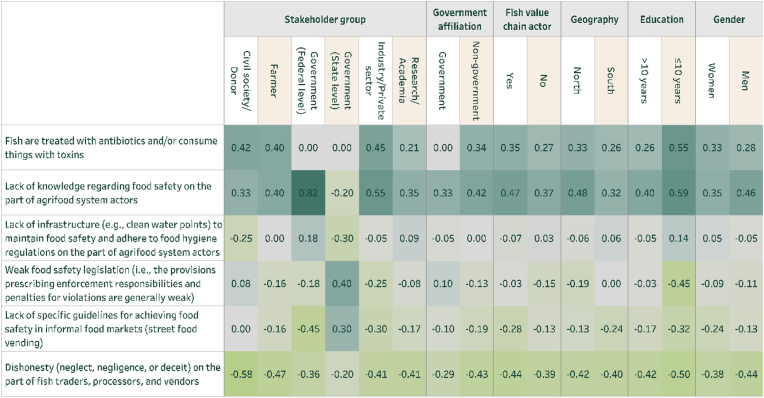
Challenges for the safety of fish (disaggregated) Note: Sample size is as in Fig. 1, with several additional categories: Civil society/donor (12); Farmer (46); Government (federal level) (12); Government (state level) (11); Industry/private sector (45); Research/academia (69); Fish value chain actor (73); Not fish value chain actor (127).

Source: RSM2SNF Nigeria Stakeholder Perceptions Survey 2022

To compare perceptions of food safety challenges for fish across different subsamples, the values were averaged within each group, resulting in a range from −1 (if all respondents in the group selected a given option as least serious) to +1 (if all selected the option as most serious). Fig. 3 shows that when comparing across stakeholder groups, it becomes apparent that a lack of knowledge regarding food safety is most commonly perceived as a key challenge by respondents from the federal government but those from the state government were most likely to fault weaknesses in legislation and a lack of guidelines for street vending as the main challenge for fish safety. This may suggest that the state government is shifting responsibility to the federal level, as the latter has an exclusive mandate over food safety regulations and guidelines even as the former is tasked with implementing them. Meanwhile, federal government representatives were more likely to also consider a lack of infrastructure (e.g., clean water points) to maintain food safety/hygiene to be a challenge. This shifts responsibility to the state level—which mostly oversees water distribution and market infrastructure—as well as to fish producers and traders.

When comparing responses of those who were and were not affiliated with government (now pooling government representatives at the federal and state levels), we find divergence when it comes to food safety practices for fish production. While non-government respondents considered use of antibiotics to be a serious food safety challenge for fish, this was ranked as relatively unimportant by government respondents. This apparent disconnect between state and non-state actors is somewhat surprising, given the relatively educated sample and recent media coverage of antibiotics use in Nigeria (Agency Report, 2021; Onwuzoo, 2021). A *t*-test confirms that the mean value assigned to this challenge is significantly different across government and non-government respondents (t = 1.81, P = 0.073).

**Fig. 4:**
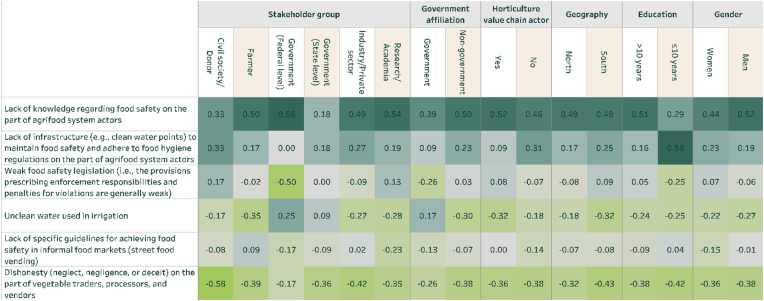
Challenges for the safety of vegetables (disaggregated) Note: Sample size is as in Fig. 3, with several additional categories: Horticulture value chain actor (91); Not horticulture value chain actor (109).

Source: RSM2SNF Nigeria Stakeholder Perceptions Survey 2022

The survey asked a parallel set of questions for vegetables (Fig. 4). As with fish, dishonesty on the part of food system actors was not regarded as a pressing challenge by any stakeholder while almost all respondents saw lack of knowledge as a challenge. Parallel to the findings for fish, federal government respondents were least likely to view weak food safety legislation as a problem. When comparing subsamples categorized by their affiliation with government, we find notable divergence in perceptions of the importance of infrastructure such as clean water points. This was the second most important challenge noted by non-government actors but was considered relatively unimportant by government respondents. Meanwhile, government representatives were significantly more likely than others to view use of unclear irrigation water as a challenge (t = 2.63, P = 0.009). When comparing those directly engaged and not engaged with the horticulture value chain, it emerges that those outside of the value chain place a heavier emphasis on a lack of infrastructure (t = 1.90, P = 0.059), and those in the value chain place very little weight on the use of unclean water for irrigation. Representatives of civil society and research tend to give more weight to weak food safety legislation, while farmers give more weight to a lack of guidelines for food safety in informal markets (though the latter differences are not statistically significant). Interestingly, and unlike the findings for fish, those with less education are most likely to see lack of infrastructure as the main challenge for the safety of vegetables, while their more educated counterparts see lack of knowledge about food safety as the main barrier.

### 5.3 Sub-group views on food affordability

**Fig. 5:**
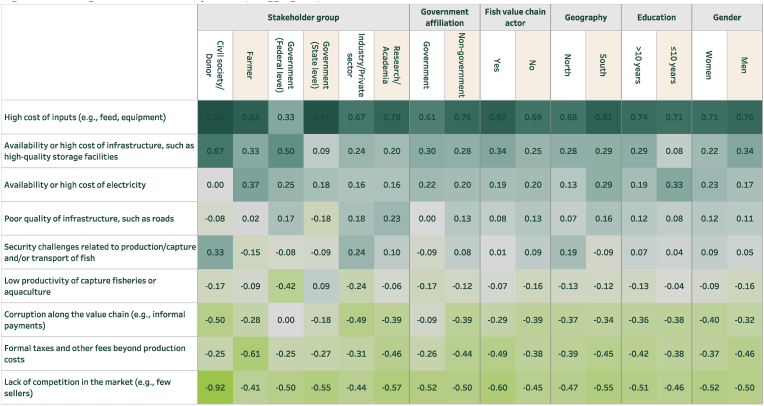
Challenges for the affordability of fish (disaggregated) Note: Sample size is as in Fig. 3.

When examining affordability, a separate set of challenges was identified. For fish (Fig. 5), there is general alignment across stakeholder groups. However, it is noteworthy that representatives of government at the federal level were least likely to view the high cost of inputs as a challenge to the affordability of fish. In addition, representatives from civil society/development partners and industry were most likely to consider security challenges to be a problem. Respondents in the south tended to view the high cost of inputs, the unavailability or high cost of electricity, and the poor quality of infrastructure to be of greater importance (relative to other challenges) than those in the north. At the same time, respondents in the north were much more likely to view security challenges related to the production/capture and/or transport of fish to be a problem (t = 2.83, P = 0.005).

**Fig. 6:**
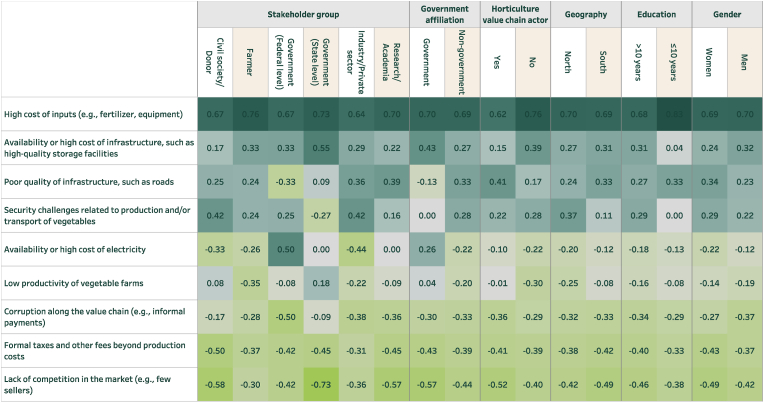
Challenges for the affordability of vegetables (disaggregated) Note: Sample size is as in Fig. 4.

For vegetables (Fig. 6), high input costs for production were identified as the greatest challenge across most stakeholders but particularly prioritized among farmers. Representatives of government at the federal level were most likely to view the availability or high cost of electricity as a meaningful challenge, though this sentiment was not shared by representatives of industry/private sector. Security challenges were identified as even more problematic for vegetables than for fish among multiple stakeholder groups. Notably though, this stands out as more prominent in the north rather than in the south. This is likely because vegetable production (e.g. tomatoes, onions, and peppers) is concentrated in the north where Boko Haram and other insurgents have exacerbated insecurity.

### 5.4 Policy spending priorities

These perceptions of challenges matter because they can sometimes inform preferences for policy solutions. To analyze this, survey respondents were also asked, “If the government could increase its spending on programs to improve the affordability and/or safety of fish (or vegetables) in Nigerian markets, which of the following areas do you think should be the highest and lowest priority for additional investment?” From a list of nine options, respondents selected the three most important (highest priority) and three least important (lowest priority) efforts.

**Fig. 7:**
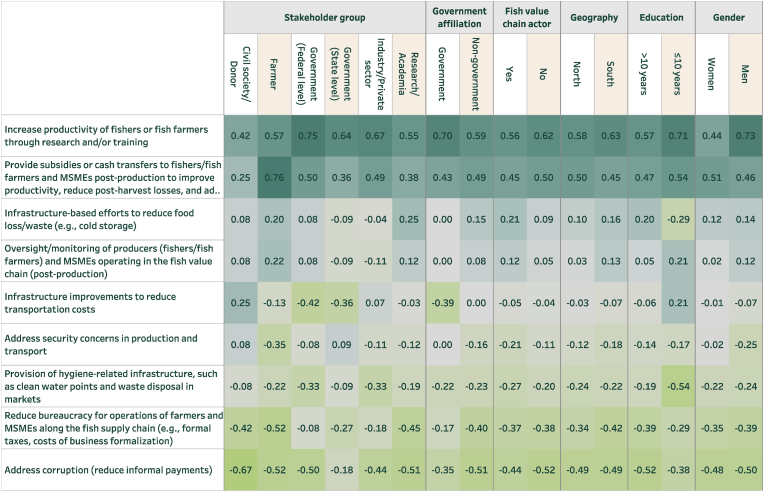
Programs to improve the affordability and/or safety of fish (disaggregated) Note: Sample size is as in Fig. 3.

**Fig. 8:**
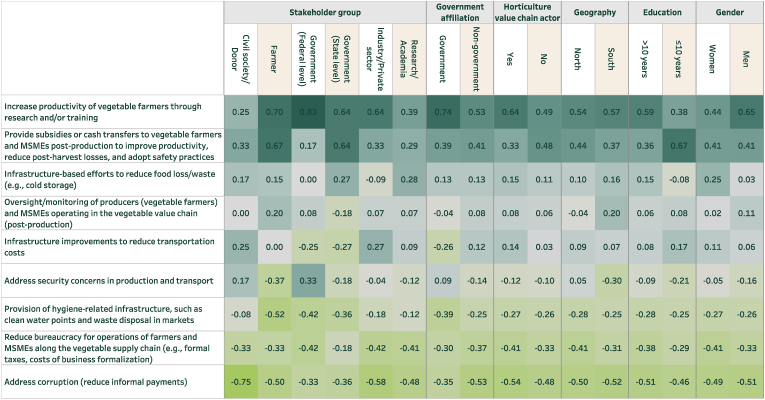
Programs to improve the affordability and/or safety of vegetables (disaggregated) Note: Sample size is as in Fig. 4.

Fig. 7, Fig. 8 reveal several trends. First, while stakeholders tend to distribute their top challenges for safety and affordability across a wide range of issues, they demonstrate greater concentration of their most preferred policy solutions. For instance, for both fish and vegetables, the most preferred program by almost all stakeholder groups for addressing affordability and safety is to improve research and training and subsidies/cash transfers. Second, stakeholders’ preferences largely follow from their beliefs about the key policy problem for both sub-sectors. For example, both fish and vegetable farmers emphasized that the high cost of inputs were a major challenge for affordability (Fig. 5, Fig. 6) and are most likely to favor receiving subsidies/cash transfers to deal with high costs (Fig. 7, Fig. 8). Third, the policy options that most align with making fish and vegetables more affordable were given more weight than those that most explicitly target food safety (e.g. provision of hygiene-related infrastructure). In a post-survey validation event in Nigeria, the preference for options that target affordability above safety was affirmed by participants.

Fourth, some issues that were prioritized as major challenges for food safety and affordability are not prioritized for policy programs. This is most notable for security concerns related to vegetable production; while identified as a major concern across most stakeholder groups in Fig. 6, especially among those in the North, it is substantively de-prioritized in Fig. 8. This may be because respondents are also considering feasibility of reforms rather than just desired interventions when forced through BWS to identify their priorities.

Some other interesting divergences also emerge. For example, when focusing on vegetables (Fig. 8), infrastructure-based efforts to reduce food loss/waste (e.g., cold storage) were de-prioritized by representatives of industry/private sector even as cold storage might be of particular use to wholesalers. At the same time, representatives of industry/private sector were more likely than most other groups to prioritize infrastructure improvements to reduce transportation costs. Across genders, women were more likely than men to prioritize infrastructure-based efforts to reduce food loss/waste (t = 2.00, P = 0.046). Across respondents that were and were not representatives of government, those with a government affiliation were less likely than others to prioritize infrastructure improvements to reduce transportation costs (t = −2.25, P = 0.025) and more likely to focus on electricity costs and availability.

## 6 Discussion

With a focus on two important sub-sectors—fish and vegetables—and disaggregating across different stakeholder groups, we uncovered both convergent and divergent policy beliefs regarding the binding constraints to addressing food safety and affordability in Nigeria. In turn, we showed how different stakeholders prioritize possible policy interventions to enhance safety and affordability. Across all groups, corruption along value chains due to informal taxes and excessive bureaucracy are not viewed as priority challenges. At the same, there is divergence in several areas, particularly when focusing on food safety. For food affordability, high costs of inputs and security were seen as common challenges across most, though not all, stakeholders for both value chains.

A key question is where do these divergent beliefs emerge? In much of the literature, education and ideology—especially preconceptions about the role of the state versus the market—are major determinants of differential policy beliefs (Béland and Cox, 2010; Blyth, 2015). While educational differences do persist in beliefs for vegetable food safety, the high average level of education across the sample implies that this is not the overriding factor. Moreover, while possible cultural differences are reflected in regional variation in fish safety perceptions, there are no clear economic ideological variations that would drive belief divergence around food safety.

Instead, our findings are suggestive of several interrelated dynamics. The first is proximity to the value chain because this affects information asymmetries in different ways. Farmers and non-farm private sector actors involved in fisheries, for example, would directly observe changes in fish appearance and quality due to toxins and antibiotics that might not be apparent to policymakers. Similarly, those farming vegetables might be more aware of a lack of contamination in irrigated water than decisionmakers in Abuja or a state capital. This reinforces the general disconnect between the government and citizens on local development issues in Nigeria more broadly (Victor, 2021). Second, drawing on Mogues and Olofinbiyi's (2020) findings, there are also asymmetries in responsibilities among policy actors for functions, and this may bias certain actors towards selecting particular constraints. For instance, state government officials who are responsible for enforcement of food safety regulations identify poor legislation and insufficient guidelines—mandates of the federal government—as among top problems for fish safety.

Third, the preferred policy options suggest that interventions aimed at increasing productivity and therefore affordability are still prioritized over safety. This resonates with other research showing that affordability concerns often trump food safety preoccupations (Liguori et al., 2022). Fourth, as found in other African countries (Mason et al., 2019), both fish and vegetable farmers prefer subsidies or cash transfers—private goods—rather than infrastructure public goods. Finally, even though security is deemed a major issue, especially for vegetable affordability, across most stakeholders and especially among the non-government sample, it is de-prioritized among non-government stakeholders when looking at policy programs. Following Kyle's (2018) observations about policy beliefs being shaped by trust about government delivery, this suggests low trust in the ability of the government to deliver security benefits and therefore, this policy option is not viewed as very feasible; in fact, public opinion assessments in Nigeria note that not only do citizens perceive insecurity is growing but also that the government has a very poor performance in tackling it (Mbaegbu and Duntoye, 2023).

Uncovering these dynamics would have been impossible without the fine-tuned disaggregation of stakeholder categories that our survey allowed or the utilization of BWS. As such, the analysis should be viewed as illustrative of the range of the potentially variegated set of policy beliefs prevailing among agrifood system stakeholders. In this way, it could prove a useful approach to identify areas of the greatest divergence where dialogue among targeted stakeholder groups could facilitate common understandings of binding constraints and opportunities. Indeed, and in line with calls for policies based on a better understanding of Nigeria's food supply chains (Liverpool-Tasie et al., 2021a), a greater willingness of government to listen to farmer and industry perspectives and participate in field visits to informal markets and places of production might help with reconciling these divergences.

## 7 Limitations

Our study faces at least two limitations. First, as noted earlier, our respondents were based on a convenience sample that included attendees to a project on the provision of safe, nutritious food in Nigeria. Although not representative of the country's fullest set of agrifood system actors, these respondents are among those with high levels of engagement in the fish, vegetable, and broader agrifood system, and they nonetheless still demonstrated considerable divergence in their policy beliefs. A broader set of stakeholders, including consumers and those less educated, would likely have resulted in an even wider variation in prioritized challenges and policy interventions. In other words, our sample likely underestimates the extent to which such divergence in beliefs exists. Secondly, some of our sub-samples are a bit limited in sample sizes. We have though ensured that any claims of statistical significance are limited to comparisons of samples that contained adequate observations in each category for such a test.

## 8 Conclusion

Food safety and affordability are major challenges facing LMICs, and this is particularly true for fresh, healthy foods that are critical for nutritional well-being but which are often out of reach for the poorest households. While food system transformation requires reforms to enhance access to safe, affordable foods (Fanzo et al., 2021), it inevitably requires making explicit trade-offs across different policy options, which an approach such as BWS helps to simulate. Moreover, it involves tracing whether those policy options reflect perceived challenges in the food system or perceived viability of reforms taking place.

This paper revealed that policy beliefs about food safety and affordability are highly variable across both different food system stakeholders and value chains. This points to the need for contextualized approaches that are informed by not only cost-benefit calculations but also experiential approaches that push decisionmakers to understand issues from the perspective of those most proximate to the issue. In doing so, future research should aim to uncover the range of factors that drive belief divergence, including information asymmetries, responsibility asymmetries, and low trust in government performance.

In the context of Nigeria, the findings of this paper can be useful for ongoing policy engagement. For instance, the 2019 National Food Safety and Quality Bill was never passed into law before parliamentarians’ tenure ended in May 2023 following elections. As such, in revisiting this Bill, there is a window of opportunity to pursue more inclusive processes that examine these divergent policy beliefs and uncover areas where there is the greatest consensus about needed reforms. Similarly, the Nigerian government declared a “state of emergency on food security” in 2023 with an 8-point agenda that involves tackling food affordability. This is likewise a critical juncture for more inclusive processes to ensure that divergent perceptions are harnessed to inform the core elements and implementation of this ambitious agenda.

## Funding and ethics clearance

This work was funded by the 10.13039/100000865Bill and Melinda Gates Foundation under a project with Funding Title: “Actionable research to support African MSMEs to supply affordable, safe, and nutritious foods.” All studies in this project involving humans were approved by 10.13039/100007709Michigan State University Institutional Review Board and were conducted in accordance with the local legislation and institutional requirements. The participants provided their written informed consent to participate in this study.

## CRediT authorship contribution statement

**Lenis Saweda O. Liverpool-Tasie:** Conceptualization, Funding acquisition, Methodology, Project administration, Supervision, Writing – original draft, Writing – review & editing. **Ayala Wineman:** Conceptualization, Formal analysis, Methodology, Project administration, Visualization, Writing – original draft, Writing – review & editing. **Danielle Resnick:** Conceptualization, Writing – original draft, Writing – review & editing.

## Declaration of competing interest

The authors have no conflicts of interest to declare.

## Data Availability

Data will be made available on request.
